# Quantity Discrimination in Domestic Rats, *Rattus norvegicus*

**DOI:** 10.3390/ani6080046

**Published:** 2016-08-03

**Authors:** Laura Cox, V. Tamara Montrose

**Affiliations:** Animal Behavior and Welfare Research Group, Department of Animal and Land Sciences, Hartpury University Centre, Hartpury, Gloucestershire GL19 3BE, UK; Laura.Cox@Hartpury.ac.uk

**Keywords:** quantity discrimination, numerical discrimination, rodent cognition, rat

## Abstract

**Simple Summary:**

Quantity discrimination involves distinguishing which of two quantities is greater. This discrimination between larger and smaller quantities has only been demonstrated in rats post extensive training. We tested whether domestic rats could perform quantity discrimination without explicit training. We found that rats could distinguish the greater amount in comparisons of 1 vs. 2, 2 vs. 3, 3 vs. 5, 3 vs. 8, 4 vs. 6, and 4 vs. 8. Rats could not distinguish between 3 vs. 4, 4 vs. 5 and 5 vs. 6. We also found that as the ratio between quantities became finer the choice of the larger quantity decreased. We conclude that rats can perform quantity discrimination without extensive training and that their quantity discrimination ability is influenced by the ratio between quantities.

**Abstract:**

Quantity discrimination is a basic form of numerical competence where an animal distinguishes which of two amounts is greater in size. Whilst quantity discrimination in rats has been investigated via training paradigms, rats’ natural quantity discrimination abilities without explicit training for a desired response have not been explored. This study investigated domestic rats’ ability to perform quantity discrimination. Domestic rats (*n* = 12) were examined for their ability to distinguish the larger amount under nine quantity comparisons. One-sample *t*-tests identified a significant preference for the larger quantity in comparisons of 1 vs. 2, 2 vs. 3, 3 vs. 5, 3 vs. 8, 4 vs. 6, and 4 vs. 8. No preference between quantities was found for comparisons of 3 vs. 4, 4 vs. 5 and 5 vs. 6. Overall, this study drew two key conclusions. Firstly, that domestic rats are capable of performing quantity discrimination without extensive training. Secondly, as subjects adhered to Weber’s law, it was concluded that the approximate number system underpins domestic rats’ ability to perform spontaneous quantity discrimination.

## 1. Introduction

The ability to discriminate between different quantities can improve survival and reproductive success in a range of contexts for instance by enabling animals to monitor and compare the number of predators, group members, food items available or competitors [1]. Quantity discrimination has been reported in a range of taxa including mammals (e.g., Dogs: [2], African elephants [3], Cats: [4], Capuchin monkeys: [5], Great apes: [6]), birds (e.g., Domestic Chicks [7] Clark’s Nutcrackers: [8], North Island robins: [9], African grey parrots: [10]), invertebrates (e.g., Japanese ants [11], Yellow mealworm beetles [12]), fish (e.g., Angelfish: [13], Mosquitofish: [14]; Redtail Splitfin [15]) and amphibians (e.g., Frogs: [16], Salamanders: [17,18]).

Quantity discrimination is the most basic type of numerical competence, since differentiation of the larger from the smaller group can occur without needing to understand the true amounts each group contains [19,20]. Two mechanisms have been presented to explain how human and non-human animals perform quantity discrimination, the object-file system and the approximate number system (also termed the analog magnitude mechanism) [21,22]. The object-file system is an indexing system reliant upon working memory and the maximal number of objects the animal has the ability to remember. Thus, the object-file system is perceived to be employed if the animal cannot distinguish between two amounts containing in excess of three to four items [22,23,24]. The approximate number system is an analog magnitude system associated with adherence to Weber’s law, where quantity discrimination ability is influenced by the ratio between quantities [22,25,26]. A crude ratio, such as 1 vs. 5 is easier to discriminate than a fine ratio, such as 4 vs. 5 [23]. Adherence with Weber’s law means that the finer the ratio between two quantities, the harder it is for the animal to differentiate between them [25,26].

Domestic rats (*Rattus norvegicus*) are a common animal model for scientific research [27,28], as well as being increasingly popular as a companion animal [29,30]. Rats have been shown to display cognitive abilities such as episodic memory [31,32], metacognition [33,34] and prospective memory [35]. Whilst quantity discrimination has been explored in rats, to our knowledge, to date rats’ ability to differentiate quantities has only been investigated utilizing extensive training paradigms involving discrimination between neutral non biologically-relevant stimuli (e.g., [36,37,38]). In these studies, rats were extensively trained (16 h [36]; 15 days [37]; 30 days [38]) to press levers in order to demonstrate discrimination between different auditory or visual sequences [36,37,38].

Findings from studies utilizing extensive training can be suggested not only to lack ecological validity, but also this prior training can improve animals’ quantity discrimination abilities to a level beyond that which would be utilized spontaneously [10,24,39,40].

The aim of this study was to examine domestic rats’ ability to perform quantity discrimination without explicit training for a desired response, such as is seen in intensive training paradigms. As part of this aim, whether rats utilize the object-file system or approximate number system when discriminating quantities was also explored.

## 2. Materials and Methods 

### 2.1. Subjects

Twelve adult companion rats participated in this study (6 males, 6 females, aged between 9 and 24 months), sampled from a private collection in Herefordshire, UK. All subjects were group-housed by sex. Each group was housed within the SAVIC**^®^** Royale 95 which consists of two levels each sized 95 cm × 63 cm, with the overall dimensions being 95 cm × 63 cm × 120 cm [41]. Rats were provided with ad lib water and fed twice daily with Pets at Home**^®^** Rat Nuggets at 9:30 a.m. and 9:30 p.m.

### 2.2. Procedure

Experimental trials were performed within the home cage of each focal rat in order to minimize anxiety and ensure engagement with the choice task. Trials took place within a designated area, sized 57.5 cm × 63 cm, located within the lower level of the cage. This section of the cage was chosen to minimize concerns regarding territoriality. Whilst the precise location of a given male rat’s territory is unclear, the territory of female rats tends to be based within the sleeping area [42]. All rats exhibited sleeping behavior in the upper level of their cages.

Each choice trial was performed individually by the focal animal with conspecifics absent from the cage. Trial sessions took place at 8:30 a.m. and 8:30 p.m., before the rats were fed, in order to promote engagement with the trials. At the start of each trial, two clear 9 cm by 9 cm petri dishes baited with the appropriate quantities of sliced peeled 1 cm^3^ apple pieces were placed into the testing area. These were 21 cm apart and the two quantities were separated by an opaque plastic panel to ensure that only one quantity could be accessed at a time. The dishes were initially sealed using transparent lids to ensure that rats did not select a dish impulsively.

The focal rat was then placed at the starting point 20 cm away from the two food quantities. The rat was left to explore the area until the location of both quantities had been accessed. This was determined by when both of the rat’s front feet had crossed a blue tape line marked on the floor of the area 5 cm around each food dish. The rat was then returned to the starting point, the lids to the dishes simultaneously removed and the rat released. The trial ended when the rat picked up or commenced consuming the apple according to which occurred first. 

To reduce issues associated with side-bias, the greater amount was situated left of the divider for 50% of trials and on the right of the divider for 50% of trials. The sequencing of the placement of the greater amount was also randomized to reduce the likelihood that rats could track left or right placement of the greater amount and select according to where it had been located in the previous trial.

Approval for the study was not needed under the Animals (Scientific Procedures) Act 1986. The study was approved and abided by the guidelines of the institutional Research Ethics Committee (ETHICS2015-32).

### 2.3. Pre-Test Condition

Before the experimental conditions, rats underwent a pre-test phase to habituate them to the procedure of selecting a quantity from one of the two dishes and obtaining the food selected. This approach where, prior to testing with the novel conditions, a pre-test condition is initially presented has been used in previous work in species such as elephants, dogs and mosquitofish [3,14,43]. This pre-test phase is important to familiarize the rats with the task and allow them to learn that only one quantity can be selected each trial without being allowed to make a second choice [3].

As the partition dividing the two quantities and the process of being moved to the testing area and placed on the starting point were novel to the subjects, habituation also prevented neophobia during experimental trials and increased the likelihood that subjects would concentrate on the specific task as opposed to any surrounding stimuli. Rats underwent the same procedure as utilized during the experimental trials, however apple was presented in a 1 vs. 5 quantity comparison. This ratio was selected as, based on Weber’s law, it was predicted to be relatively simple to differentiate [43]. This process was repeated for each rat until the criterion of selecting the greater quantity in four consecutive trials was achieved. 

### 2.4. Quantity Comparison Conditions

Each rat experienced nine quantity comparisons, 1 vs. 2, 2 vs. 3, 3 vs. 4, 3 vs. 5, 3 vs. 8, 4 vs. 5, 4 vs. 6, 4 vs. 8 and 5 vs. 6. All subjects performed each condition four times. Under naturalistic choice paradigms, it is predicted that the larger quantity will be chosen as this enables acquisition of the greatest amount of food and therefore is the most evolutionarily favorable selection [24,25,43]. Thus whether rats selected the greater amount more frequently was the measure used to determine their ability to discriminate between the two quantities. These nine quantity combinations varied in the fineness of the ratio and provided ratios from 3 vs. 8 (crude ratio) to 5 vs. 6 (fine ratio), offering insight into whether rats’ discrimination ability followed Weber’s law. In addition, the comparisons, 5 vs. 6, 4 vs. 8 and 4 vs. 6, and potentially also the comparison 4 vs. 5, exceeded the maximum number of three to four items which most animals may differentiate between using the object-file system [22,23,24].

### 2.5. Volume, Surface Area and Olfactory Control Conditions 

Quantity tends to vary with other non-numerical cues such as surface area, volume or olfactory cues [4,14,39]. Three control conditions were therefore included to determine whether rats selected according to volume, surface area occupied by the two quantities or olfaction respectively. As for the experimental conditions all subjects performed each condition four times.

The volume control condition determined whether selections were made according to differences in volume. Rats were offered a selection between one dish containing two pieces of 1 cm^3^ apple and one dish containing a single piece of apple sized 2 cm^3^. One dish therefore offered a larger number of food items than the alternative option but the same absolute volume of food. A significant preference for two single pieces would suggest that subjects consider the number of items comprising each quantity to determine the larger amount, whilst random choice would suggest that volume is being considered [24]. Two pieces vs. one large piece were utilized as these amounts are within the object-file system’s capacity. 

In the surface area control condition, rats were presented with one dish containing two pieces of 1 cm^3^ apple positioned 5 mm apart and a second dish containing two pieces of 1 cm^3^ apple positioned 3 cm apart. This condition examined whether rats selected according to the total surface area outlined by the perimeter of the apple pieces as the option containing apple pieces 3 cm apart provided the same amount of food as the alternative option however covered a greater surface area on the dish [8]. 

The olfactory control condition examined whether rats selected utilizing olfactory cues. Following previous studies (e.g., [2,44]), rats were offered two quantities with a 6:1 ratio. These were measured according to weight (12 g vs. 2 g) and placed within opaque Petri dishes. The dishes were suspended 15 cm above the floor of the cage, thus ensuring the dishes were above the rats’ line of sight but not beyond their reach. The dishes were open to ensure parity in the release of odor cues to all other conditions. In this olfactory control condition the rats were thus unable to see the differing amounts of food but were able to smell and reach them.

### 2.6. Data Analysis

Each subject performed each condition four times. The number of choices each rat made for the larger quantity was determined for each quantity comparison condition. One-sample *t*-tests were then performed to determine if the number of choices for the larger quantity was significantly different than would be expected by chance. This was performed for each experimental condition and to examine which option was chosen in the control conditions. A Spearman rank-order correlation test was then used to examine if there was a relationship between the ratio of the large and small quantities and the proportion of choices for the larger of the two quantities. All analyses were carried out in SPSS (version 22.0, SPSS Inc., Armonk, NY, USA, 2013).

## 3. Results

### 3.1. Quantity Comparison Conditions

The rats showed a significant preference for the greater quantity in the comparisons 1 vs. 2, 2 vs. 3, 3 vs. 5, 3 vs. 8, 4 vs. 6 and 4 vs. 8. No significant preference for the larger quantity was shown in comparisons of 3 vs. 4, 4 vs. 5 and 5 vs. 6 (Table 1).

Spearman rank-order correlation analysis found that as the fineness of the ratio between the large and small quantities increased (e.g., from 3:8 to 5:6) this was weakly associated with a decrease in the proportion of choices for the larger of the two quantities (r_s_ = −0.385, *N* = 108, *p* < 0.0001).

### 3.2. Volume, Surface Area and Olfactory Control Conditions

No significant difference was found between the selection of the two pieces of 1 cm^3^ apple or the single piece of apple sized 2 cm^3^ (t(11) = 0.000, *p* = 1.000, mean number of trials where two small pieces were chosen = 2.00, standard error of the mean (SEM) = 0.369). In the surface area control condition, rats showed a significant preference for the two 1 cm^3^ food items spaced 5 mm apart rather than the two food items spaced 3 cm apart (t(11) = −2.462, *p* = 0.032, mean number of trials where 3 cm apart items were chosen = 1.25, SEM = 0.305). No significant difference was found between selection of the small and large quantities in the olfactory control condition (t(11) = −0.364, *p* = 0.723; mean number of trials where greater quantity chosen = 1.92, SEM = 0.229) suggesting that the rats were not discriminating between quantities of food based on olfactory cues. 

## 4. Discussion

The aim of this study was to examine whether domestic rats perform quantity discrimination without explicit training for a desired response, such as is seen in intensive training paradigms. Significant preferences for the larger quantity were displayed by the rats when presented with quantity comparisons of 1 vs. 2, 2 vs. 3, 3 vs. 5, 3 vs. 8, 4 vs. 6 and 4 vs. 8, but not when presented with comparisons of 3 vs. 4, 4 vs. 5 and 5 vs. 6.

This study also explored whether rats utilize the object-file system or approximate number system when discriminating quantities. One key indicator that animals are utilizing the object-file system to perform quantity discrimination is an inability to differentiate two quantities if one or both amounts contains more than three or four items [22,23,45]. If subjects employ the approximate number system, the ability to distinguish two amounts is influenced by the ratio between them. According to Weber’s law, the finer the ratio between two quantities, the harder it is for the animal to differentiate between them [23,25,26]. Whilst there are many studies in a range of taxa evidencing use of the approximate number system to perform quantity discrimination (e.g., [5,7,10,13,23,43,44,46]), use of the object-file system has also been described in a variety of species including humans [22], rhesus macaques [24], domestic chicks [47], horses [48], amphibians [16,18] and mosquitofish [49]. In this study the negative correlation found between the fineness of the ratio and the proportion of trials where the larger quantity was selected indicates that the rats’ ability to differentiate the two amounts decreased as the ratio became finer (e.g., from 3:8 to 5:6). This suggests that rats adhered to Weber’s law and hence utilized the approximate number system when performing quantity discrimination. The rats’ failure in the quantity discrimination trials to distinguish the larger amount when presented with comparisons of 3 vs. 4, 4 vs. 5 and 5 vs. 6 also corresponds with this interpretation. Adherence with Weber’s law enables differentiation between two high numerical amounts as easily as two low amounts, provided the ratio between the amounts remains constant [25,26]. This adherence with Weber’s Law is indicated by the rats’ ability to discriminate between the two amounts when the ratio between quantities was 1:2 and 2:3, for both lower (1 vs. 2, 2 vs. 3) and higher (4 vs. 8, 4 vs. 6) values. Whilst rats’ success at discriminating between quantity comparisons of 1 vs. 2, and 2 vs. 3, but inability to differentiate 3 vs. 4, 4 vs. 5 or 5 vs. 6, could also be explained via use of the object-file system, the rats’ success when considering 4 vs. 8 and 4 vs. 6 does not support this interpretation. Overall, these findings are suggestive that quantity discrimination in rats abides by Weber’s Law, and thus that rats employ the approximate number system when performing quantity discrimination.

A key consideration in studies of quantity discrimination is ensuring that the findings cannot be explained by the species use of non-numerical cues [39,47,50]. In this study control conditions for olfactory cues, surface area and volume were utilized. During the olfactory control condition, where olfactory but not visual information was available, rats did not show a significant preference for the larger amount suggesting rats were not utilizing odor to detect the greater quantity. Whilst rats can use their olfactory senses to track food [42,51], these findings suggest that olfactory cues do not provide rats with enough information to allow quantity discrimination to occur. Similar findings have been observed in other species that possess refined olfactory systems (e.g., [2,43,44]). In the surface area control, rats demonstrated a significant preference for the two items spaced 5 mm apart suggesting that if rats rely on total surface area during quantity discrimination, quantities covering the least surface area are favored. This is surprising as if surface area is used in the discrimination of food quantities, natural selection should favor animals that select the quantity covering the larger surface area. In addition, the results of the quantity discrimination trials did not support a preference for lower surface area. Thus, results of this control could suggest that rats were unlikely to be utilizing surface area to make their choice when determining the larger quantity. However, it is important to note that this control investigated the total surface area outlined by the perimeter of the apple pieces, rather than the cumulative surface area of the food per se. It is important to consider whether this control actually controlled for surface area or whether it controlled for the effects of dispersion of the food pieces. The differing densities of the two sets can also be problematic when controlling for this factor [39]. Another factor of relevance to this control is contour length [47,52]. Contour length can be an important non-numerical cue when discriminating between quantities [52]. Since both sets of items in the total surface area control had the same total contour length (sum total of the perimeters of the objects within each set [52]), this may have impacted on the findings for this control. Considering a total surface area control alongside approaches to investigating cumulative surface area such as using differently shaped pieces of apple as well as controls for contour length and density would be of value for future study. In the volume control condition, where rats were presented with two options comprising equal volumes but different numerical quantities, rats did not show a significant preference for either option, thus suggesting that volume of food present was considered rather than numerical information. However, during this condition it is important to note that there was the same overall amount of food in both options. Natural selection has likely selected for maximizing the overall amount of food obtained rather than the specific number of food items [39]. Since rats obtained the same energetic benefits regardless of their choice, there was little motivation to select the option containing the greatest absolute number of items. That rats in the current study selected according to volume in this control condition therefore does not confirm that rats lack the potential to utilize quantity information in other contexts. 

This study provides the first evidence, to our knowledge, that domestic rats are capable of performing quantity discrimination without explicit training and do so utilizing the same approximate number system mechanism that is utilized for quantity discrimination by human and non-human primates [5,23,53,54], parrots [10], corvids [55,56], domestic chicks [7], elephants [3], canids [43,44] and fish [13,57].

Limitations to the study include the sample size, the possibility that rats are discriminating between quantities using other non-numerical cues, such as contour length, which were not controlled for in this current study, as well as the constraints associated with attempting to control for the use of non-numerical cues in quantity discrimination studies [39], and the potential influence of learning effects due to the initial trialing with small quantities which may have enhanced the subjects’ later performance when presented with higher quantities. Future research addressing these limitations, as well as exploring related measures such as the rats’ latency to make a selection would be of value. In addition, work exploring the influence of age upon quantity discrimination in rats would be of relevance. Age-related differences in quantity discrimination ability have been observed in species such as guppies, humans and gorillas [40,54,58] and are of value in elucidating the development of numerical competence. Rats also provide a good model for exploring the effects of domestication upon quantity discrimination abilities. The current study’s findings would facilitate comparisons between quantity discrimination abilities in domestic and free-living rats, similarly to those that have been made between domestic and non-domestic canids [2,59]. 

## 5. Conclusions

The preference for the larger quantity shown by the rats when presented with quantity comparisons of 1 vs. 2, 2 vs. 3, 3 vs. 5, 3 vs. 8, 4 vs. 6 and 4 vs. 8 indicates that domestic rats are able to perform quantity discrimination without explicit training. The rats’ selection of the larger amount also decreased as the ratio between quantities became finer. This suggests that quantity discrimination in rats adheres to Weber’s Law, and that rats employ the approximate number system when performing quantity discrimination. Future research could expand on the findings of this study by investigating the effects of age and domestication upon quantity discrimination abilities in this species.

## Figures and Tables

**Table 1:** Analysis showing effects of quantity comparison on choice of the greater quantity.

Quantity Comparison	Ratio	Mean Number of Trials Where Greater Quantity Chosen	Standard Error of the Mean (SEM)	*t*	d.f	*p*
1 vs. 2	1:2	3.08	0.193	5.613	11	<0.0001
2 vs. 3	2:3	3.08	0.260	4.168	11	0.002
3 vs. 4	3:4	2.67	0.310	2.152	11	0.054
3 vs. 5	3:5	3.00	0.213	4.690	11	0.001
3 vs. 8	3:8	3.58	0.149	10.652	11	<0.0001
4 vs. 5	4:5	2.00	0.302	0.000	11	1.000
4 vs. 6	2:3	2.83	0.271	3.079	11	0.010
4 vs. 8	1:2	2.75	0.329	2.283	11	0.043
5 vs. 6	5:6	2.08	0.336	0.248	11	0.809
